# Supplementary material for: Achieving cervical cancer elimination: The simulated impacts of HPV vaccination and transitioning from liquid-based cytology to HPV-based screening test
Source: PLoS One. 2024 Jul 25;19(7):e0307880. doi: 10.1371/journal.pone.0307880 (PMC11271949; doi:10.1371/journal.pone.0307880)
Supplement: S1 File — (PDF) [file pone.0307880.s001.pdf]

Appendix A: List of parameters and sources

Parameter	Description	Final/Calibrated value	Initial value	Reference
**Population**				
Total Female Population	Total female population in the year 1990 (start of simulation)		12,817,700	(1)
Birth rate (female)	Birth rate starting from the year 1990	y = 20.196e-0.015x (incurred by 10%)		(1)
Growth rate (female)				
age group 0 (0-13 years)			4,931,460	(1)
age group 1 (14 years old)	Percentage from group 0 moving to group 1 in a year	0.0769	247,040	(1)
age group 2 (15 - 19 years old)	Percentage from group 1 moving to group 2 in a year	1	1,151,100	(1)
age group 3 (20 - 29 years old)	Percentage from group 2 moving to group 3 in a year	0.2	1,984,700	(1)
age group 4 (30 - 39 years old)	Percentage from group 3 moving to group 4 in a year	0.1	1,723,300	(1)
age group 5 (40 - 49) years old)	Percentage from group 4 moving to group 5 in a year	0.1	1,286,700	(1)
age group 6 (50 - 65 years old)	Percentage from group 5 moving to group 6 in a year	0.1	1,036,240	(1)
age group 7 (>65 years old)	Percentage from group 6 moving to group 7 in a year	0. 0667	457,160	(1)
Death rate (female)				
age group 0 (0-13 years)	Death rate in 2001[a] for age group 0-13	Random uniform (0.00064, 0.00071)		(1)
age group 1 (14 years old)	Death rate in 2001* for age group 14	y = -4e-06x + 0.0003		(1)
age group 2 (15 - 19 years old)	Death rate in 2001* for age group 15-19	y = -5e-06x + 0.0004		(1)
age group 3 (20 - 29 years old)	Death rate in 2001* for age group 20-29	y = -7e-06x + 0.0005		(1)
age group 4 (30 - 39 years old)	Death rate in 2001* for age group 30-39	y = -4e-06x + 0.0015		(1)
age group 5 (40 - 49) years old)	Death rate in 2001* for age group 40-49			(1)
age group 6 (50 - 65 years old)	Death rate in 2001* for age group 50-65	y = -6e-05x + 0.0079		(1)
age group 7 (>65 years old)	Death rate in 2001* for age group above 65	y = -0.0006x + 0.0533		(1)
**Vaccination**				
Vaccination coverage	Percentage of girls age 14 years old who completed vaccination the year before (i.e at age 13)	0.9		(2, 3)
	Percentage dropped out from secondary school - after 2010<br>Adolescent out of school, female (lower secondary school age)	0.1		(4)
Vaccine Efficacy	Gardasil-4 (Quadrivalent vaccine)	100%, lifelong protection		(5)
**Susceptibility**				
Susceptibility age group 3 (20 - 29 years old)	Percentage of female population who are susceptible to be infected with HPV	RANDOM UNIFORM (0.76, 0.83)		(6-8)
Susceptibility age group 4 (30 - 39 years old)		RANDOM UNIFORM (0.76, 0.83)		(6-8)

Parameter	Description	Final/Calibrated value	Initial value	Reference
Susceptibility age group 5 (40 - 49) years old)		RANDOM UNIFORM (0.76, 0.83)		(6-8)
Susceptibility age group 6 (50 - 65 years old)		RANDOM UNIFORM (0.76, 0.83)		(6-8)
Susceptibility age group 7 (>65 years old)		RANDOM UNIFORM (0.76, 0.83)		(6-8)
**Screening**				
Screening percentage (age group)				
% screened age group 3	Percentage screened for HPV infection for age group 20 -29	0		BPKK estimation (9)
% screened age group 4	Percentage screened for HPV infection for age group 30 -39	25% over 3 years (for Pap smear)		
% screened age group 5	Percentage screened for HPV infection for age group 40 -49	25% over 3 years (for Pap smear)		
% screened age group 6	Percentage screened for HPV infection for age group 50 - 65	25% over 3 years (for Pap smear)		
% screened age group 7	Percentage screened for HPV infection for age group above 65	0		
Distribution				
distribution age group 3	Percentage of attending screening among age group 20 - 29 from all women attending screening at KK	25%	587	(10)
distribution age group 4	Percentage of attending screening among age group 30 -39 from all women attending screening at KK	25%	21,478	(10)
distribution age group 5	Percentage of attending screening among age group 40 -49 from all women attending screening at KK	25%	11,073	(10)
distribution age group 6	Percentage of attending screening among age group 50 - 65 from all women attending screening at KK	25%	3,600	(10)
Cervical cancer staging				
Stage 1	Percentage diagnosed at stage 1 among all women with cervical ca	23.7		(11)
Stage 2	Percentage diagnosed at stage 2 among all women with cervical ca	35.3		(11)
Stage 3	Percentage diagnosed at stage 3 among all women with cervical ca	22.3		(11)
Stage 4	Percentage diagnosed at stage 4 among all women with cervical ca	18.7		(11)
Sensitivity and Specificity				
HPV testing	Sensitivity dan specificity for HPV testing	Sensitivity = 97.6<br>Specificity = 94.4		(12-15)
Pap Smear (cytology)	Sensitivity dan specificity for Pap smear	Sensitivity =<br>RANDOM UNIFORM (0.71, 0.75, 10)<br>Specificity = 0.975		(12-15)
Follow-up rate				
% follow-up	Percentage follow-up after positive screening result (both Pap smear and HPV)	RANDOM UNIFORM (0.8, 0.9)		BPKK estimation (9)
**HPV Infection Rate**				

Parameter	Description	Final/Calibrated value	Initial value	Reference
age group 3	Percentage infected by HPV for age group 20 -29<br>*Average duration for HPV infection is 2 years before it clears	0.06 (6%)	107308	(10, 16)
age group 4	Percentage infected by HPV for age group 30 - 39	0.056 (5.6%)	77938	(10, 16)
age group 5	Percentage infected by HPV for age group 40 - 49	0.058 (5.8%)	59393	(10, 16)
age group 6	Percentage infected by HPV for age group 50 - 65	0.058 (5.8%)	55625	(10, 16)
age group 7	Percentage infected by HPV for age group 65 and above	0.024 (2.4%)	0	(10, 16)
**Progression**				
Progression for precancerous				
RCIN1-CIN3	Progression rate from CIN1 to CIN3	0.01		(17, 18)
RCIN2-CIN1	Regression rate from CIN2 to CIN1	0.36		(17, 18)
RCIN2-HPV	Regression rate from CIN2 to HPV	0.01901		(17, 18)
RCIN3-CIN2	Regression rate from CIN3 to CIN2	0.03		(17, 18)
RHPV-CIN1	Progression rate from HPV to CIN1	0.0829		(17, 18)
RHPV-CIN2	Progression rate from HPV to CIN2	0.0129		(17, 18)
R1 CIN2-CIN3	Progression rate from CIN1 to CIN3	0.03		(17, 18)
R1CIN1-CIN2	Progression rate from CIN1 to CIN2	0.02		(17, 18)
R1CIN1-sus	Progression rate from CIN1 to susceptible	0.25		(17, 18)
R1CIN3-HPV	Regression rate from CIN3 to HPV	0.007		(17, 18)
R1HPV-sus	Regression rate from HPV to susceptible	0.75		(17, 18)
R2CIN1-CIN2	Progression rate from CIN1 to CIN2	0.14		(17, 18)
R2CIN1-HPV	Regression rate from CIN1 to HPV	0.02		(17, 18)
R2CIN1SUS	Regression rate from CIN1 to susceptible	0.12		(17, 18)
R2CIN2-CIN3	Progression rate from CIN2 to CIN3	0.07		(17, 18)
R2HPV-SUS	Regression rate from HPV to susceptible	0.413		(17, 18)
R3CIN2-CIN3	Progression rate from CIN2 to CIN3	0.100069		(17, 18)
Progression from precancerous to cancer				
Rate of progression	Progression rate from precancerous to cancer	0.036 (for all age groups)		(17, 19, 20)
Progression at cancer staging				
Progression from Cancer Stage 1 to Stage 2	Annual transition probability from stage 1 to stage 2	0.8		(17, 19, 20)
Progression from Cancer Stage 2 to Stage 3	Annual transition probability from stage 2 to stage 3	0.8		(17, 19, 20)
Progression from Cancer Stage 3 to Stage 4	Annual transition probability from stage 3 to stage 4	0.7		(17, 19, 20)

Parameter	Description	Final/Calibrated value	Initial value	Reference
Duration of progression				
Negative PAP smear to susceptible	all age	yearly transition		(21)
Negative HPV to susceptible	all age	yearly transition		(21)
**Survival/Death Rate**				
Survival rate				
Stage 1	0 - 1 year	0.943		(22)
	1 - 2 years	0.8664		(22)
	2 - 3 years	0.8049		(22)
	3 - 4 years	0.7693		(22)
	4 - 5 years	0.7529		(22)
Stage 2	0 - 1 year	0.8543		(22)
	1 - 2 years	0.7031		(22)
	2 - 3 years	0.6005		(22)
	3 - 4 years	0.5506		(22)
	4 - 5 years	0.5226		(22)
Stage 3	0 - 1 year	0.6913		(22)
	1 - 2 years	0.496		(22)
	2 - 3 years	0.3838		(22)
	3 - 4 years	0.3447		(22)
	4 - 5 years	0.321		(22)
Stage 4	0 - 1 year	0.5299		(22)
	1 - 2 years	0.3438		(22)
	2 - 3 years	0.2648		(22)
	3 - 4 years	0.2307		(22)
	4 - 5 years	0.2302		(22)
Death rate (opposite of survival rate)				

[a] (no death rate 2000)

BPKK estimation * This is an estimation. We know that the country's current screening rate is 25%. Although 25% actually includes those who have already developed cancer, the percentage for cancer detection is small (<1%). Therefore, in this model, the 25% is assumed to be screening and detection at HPV infection/precancerous stage. The 25% rate is then divided among the 4 age groups (20 - 65) based on distribution of women attending screening by BPKK screening registry

List of sources

1. Department of Statistics Malaysia (DOSM). Population by Age Group, Sex and Ethnic Group, Malaysia [Available from: https://www.data.gov.my/data/ms_MY/dataset/population-by-age-group-sex-and-ethnic-group-malaysia.
2. Muhamad NA, Buang SN, Jaafar S, Jais R, Tan PS, Mustapha N, et al. Achieving high uptake of human papillomavirus vaccination in Malaysia through school-based vaccination programme. BMC Public Health. 2018;18:1-9.
3. World Health Organization (WHO). Human Papillomavirus (HPV) vaccination coverage [Available from: https://immunizationdata.who.int/pages/coverage/hpv.html?CODE=MYS&ANTIGEN=&YEAR=.
4. World Health Organization (WHO). School enrollment, secondary, female (% net) - Malaysia [Available from: https://data.worldbank.org/indicator/SE.SEC.NENR.FE?locations=MY.
5. Wei L, Xie X, Liu J, Zhao Y, Chen W, Zhao C, et al. Efficacy of quadrivalent human papillomavirus vaccine against persistent infection and genital disease in Chinese women: a randomized, placebo-controlled trial with 78-month follow-up. Vaccine. 2019;37(27):3617-24.
6. Institute for Public Health & Institute for Health Systems Research. National Health and Morbidity Survey 2011 (NHMS 2011). Vol. 3: Healthcare Demand. Kuala Lumpur: Ministry of Health Malaysia; 2011.
7. Institute for Public Health & Institute for Health Systems Research. National Health and Morbidity Survey 2015 (NHMS 2015). Vol. 3: Healthcare Demand. Kuala Lumpur: Ministry of Health Malaysia; 2015.
8. Institute for Public Health & Institute for Health Systems Research, . National Health and Morbidity Survey 2019 (NHMS 2019). Vol. 3: Healthcare Demand. Kuala Lumpur: Ministry of Health Malaysia; 2019.
9. Public Health Specialist Family Health Development Division (Ministry of Health Malaysia). Percentage of HPV screening in Malaysia. 2023.
10. Family Health Development Division (MOH). HPV registry 2019-2022. Putrajaya, Malaysia.
11. National Cancer Institute (NCI). Malaysia National Cancer Registry Report (MNCR) 2012-2016. Putrajaya, Malaysia: Ministry of Health Malaysia; June 2019 12 December 2022.
12. Arbyn M, Ronco G, Anttila A, Meijer CJLM, Poljak M, Ogilvie G, et al. Evidence Regarding Human Papillomavirus Testing in Secondary Prevention of Cervical Cancer. Vaccine. 2012;30:F88-F99.
13. Arbyn M, Smith SB, Temin S, Sultana F, Castle P. Detecting cervical precancer and reaching underscreened women by using HPV testing on self samples: updated meta-analyses. Bmj. 2018;363.
14. Arbyn M, Bergeron C, Klinkhamer P, Martin-Hirsch P, Siebers AG, Bulten J. Liquid compared with conventional cervical cytology: a systematic review and meta-analysis. Obstetrics & Gynecology. 2008;111(1):167-77.
15. Koliopoulos G, Nyaga VN, Santesso N, Bryant A, Martin-Hirsch PP, Mustafa RA, et al. Cytology versus HPV testing for cervical cancer screening in the general population. Cochrane database of systematic reviews. 2017(8).
16. Schiffman M, Castle PE, Jeronimo J, Rodriguez AC, Wacholder S. Human papillomavirus and cervical cancer. The lancet. 2007;370(9590):890-907.
17. Myers ER, McCrory DC, Nanda K, Bastian L, Matchar DB. Mathematical model for the natural history of human papillomavirus infection and cervical carcinogenesis. American journal of epidemiology. 2000;151(12):1158-71.

18. Canfell K, Barnabas R, Patnick J, Beral V. The predicted effect of changes in cervical screening practice in the UK: results from a modelling study. British journal of cancer. 2004;91(3):530-6.
19. Goldie SJ, Grima D, Kohli M, Wright TC, Weinstein M, Franco E. A comprehensive natural history model of HPV infection and cervical cancer to estimate the clinical impact of a prophylactic HPV-16/18 vaccine. International Journal of cancer. 2003;106(6):896-904.
20. Chauhan AS, Prinja S, Srinivasan R, Rai B, Malliga J, Jyani G, et al. Cost effectiveness of strategies for cervical cancer prevention in India. PLoS One. 2020;15(9):e0238291.
21. WHO guideline for screening and treatment of cervical pre-cancer lesions for cervical cancer prevention. Geneva: World Health Organization; 2021. Licence: CC BY-NC-SA 3.0 IGO.
22. National Cancer Registry, National Cancer Institute Ministry of Health Malaysia,. Malaysian Study on Cancer Survival (MySCan). 2018.
