# Supplementary material for: Achieving cervical cancer elimination: The simulated impacts of HPV vaccination and transitioning from liquid-based cytology to HPV-based screening test
Source: PLoS One. 2024 Jul 25;19(7):e0307880. doi: 10.1371/journal.pone.0307880 (PMC11271949; doi:10.1371/journal.pone.0307880)
Supplement: S2 File — (PDF) [file pone.0307880.s002.pdf]

Appendix B: HPV-FRAME reporting standard

Table A1: HPV-FRAME reporting standard, including core reporting standards, writing standards for HPV vaccination, the model of integrated HPV vaccination and cervical screening, and the model for LMICs, according to Canfell et al., 2019.

a) Inputs	Reported? (Y/N)	Reported by age? (Y/N)	Comments
**Core reporting standards**			
The target population for intervention	Y	Y	Vaccination (current): females aged 13 years.<br>Screening at ages 30 – 65.<br>Cancer treatment (current): all ages.
Sexual behaviour	Y	N	Susceptibility, defined as ever being married or cohabiting with a partner, was used as a proxy for sexual behaviour.<br>Data come from the National Health Morbidity Survey (NHMS): percentage ever married or have a partner.
Cohort examined for evaluation/time horizon.	Y	Y	Simulation runs from 1990 – 2090.<br>Age-standardised rates and resource utilisation reported over 2010 – 2090.<br>Cumulative cases and cases averted were reported in 2023 – 2070.<br>Overall screening cost reported over 2023 – 2070.
Quality of life assumptions	N	N	Not part of the study objectives.
Calibration	Y	Y	Parameters:<br>- Population data<br>- HPV infection prevalence<br>- Number of women screened<br>- Cancer staging
Validation	Y	Y	- National age-standardised cancer incidence<br>- Global cancer incidence<br>- Cancer incidence by age group<br>- Cancer cases
Costs	Y	N	Costs sourced from local data
**Reporting standards for HPV vaccination in adolescent individuals**			
Vaccine uptake	Y	Y	Based on the Family Health Development Division, Ministry of Health Malaysia registry.

Vaccine efficacy	Y	N	Based on Gardasil 4: 100% efficacy against HPV 16/18/6/11
Vaccine cross-protection	Y	N	Based on Gardasil 4, Cross-protection for other high-risk strains was low.
Duration of vaccine protection and waning	Y	N	Lifelong
Vaccine and delivery costs	Y	N	Included in the model, but the calculation is reported elsewhere
Pre-vaccination disease burden (including population-attributable fractions for HPV)	Y	N	Simulation runs beginning in 1990. HPV vaccination was introduced in 2010 in Malaysia
**Reporting standards for the model of cervical screening**			
Routine screening behaviour (routine and follow-up and test-of-cure)	Y	Y	Based on the report: Guidelines for Primary HPV Testing In Cervical Cancer Screening in Malaysia
Screening test(s) and colposcopy accuracies	Y	N	Sources for sensitivities and specificities of HPV test and Pap Smear are reported in the appendix. Assumes to be independent of age.
Abnormal test management (primary and triage)	Y	N	Assumed 100% treatment efficacy and coverage.
Diagnostic follow-up of abnormal tests	Y	N	Assumed 100% treatment efficacy and coverage.
Management by disease grade (confirmed disease)	Y	N	Assumed 100% treatment efficacy and coverage.
Sources of information for screening structure and parameterisation	Y	N	The screening pathways were informed by experts and compared to the literature.
**Reporting standards for integrated models of HPV vaccination and cervical screening**			
HPV type incidence, clearance and progression rates	Y	Y	Type-specific HPV incidence, clearance and progression were modelled based on HPV 16/18 and other oncogenic nonavalent.
Herd effect	Y	Y	High vaccination coverage in Malaysia (90%) contributes almost 100% reduction in HPV prevalence.
Association between vaccination and screening uptake	Y	Y	Vaccine and screening uptake were assumed to be independent of one another.
Screening test(s) and colposcopy accuracies	Y	N	Sources for sensitivities and specificities of HPV test and Pap Smear are reported in the appendix. Assumes to be independent of age.
Fixed–variable costs*	Y	N	Included in the model, but the calculation is reported elsewhere.
**Reporting standards for models of HPV prevention in LMICs#**			

HIV prevalence rates, if endemic in the country	Y	Y	Data availability of Family Health Development Division, Ministry of Health Malaysia registry
Description of any opportunistic or pilot/demonstration screening projects ongoing	Y	Y	Family Health Development Division, Ministry of Health Malaysia National Screening Plan: Guidelines for Primary HPV Testing In Cervical Cancer Screening in Malaysia
Costs	Y	N	Included in the model, but the calculation is reported elsewhere.
**b) Outputs**	**Reported? (Y/N)**	**Reported by age? (Y/N)**	**Comments**
**Core reporting standards**			
Cancer incidence, mortality, life years, QALYs/DALYs (as appropriate)	Y	Y	Age-standardized and age-specific incidence and mortality rates were reported.
HPV prevalence, pre-intervention	N	N	Data not available.
CIN2 detected	N	N	Data not available.
Sensitivity analysis on key inputs	N	N	Data not available.
Incremental cost-effectiveness ratios and costs saved	Y	N	Included in the model, but the calculation is reported elsewhere.
**Reporting standards for HPV vaccination in adolescent individuals**			
Absolute reductions in HPV infections and/or warts post-vaccination	N	N	While the reduction of HPV infection was modelled and the findings are available, they are not reported in the outcome since the focus was on reducing cervical cancer incidence.
Absolute reductions in CIN2+ post-vaccination	N	N	While the reduction of CIN2+ post-vaccination was modelled and the findings are available, they are not reported in the outcome since the focus was on the decrease in cervical cancer incidence.
Absolute reductions in invasive cancer (cervical and other HPV cancers, as relevant) post-vaccination	Y	Y	Cervical cancers only (boundary of the model).

Source: Karen Canfell, Jane J. Kim, Shalini Kulasingam, Johannes Berkhof, Ruanne Barnabas, Johannes A. Bogaards, Nicole Campos, Chloe Jennett, Monisha Sharma, Kate T. Simms, Megan A. Smith, Louiza S. Velentzis, Marc Brisson, Mark Jit. HPV-FRAME: A consensus statement and quality framework for modelled evaluations of HPV-related cancer control, Papillomavirus Research, Volume 8, 2019, 100184, ISSN 2405-8521, https://doi.org/10.1016/j.pvr.2019.100184.
