# Supplementary material for: Achieving cervical cancer elimination: The simulated impacts of HPV vaccination and transitioning from liquid-based cytology to HPV-based screening test
Source: PLoS One. 2024 Jul 25;19(7):e0307880. doi: 10.1371/journal.pone.0307880 (PMC11271949; doi:10.1371/journal.pone.0307880)
Supplement: S3 File — (PDF) [file pone.0307880.s003.pdf]

Appendix C: Validity of model

a) Validation: Cancer distribution by age group

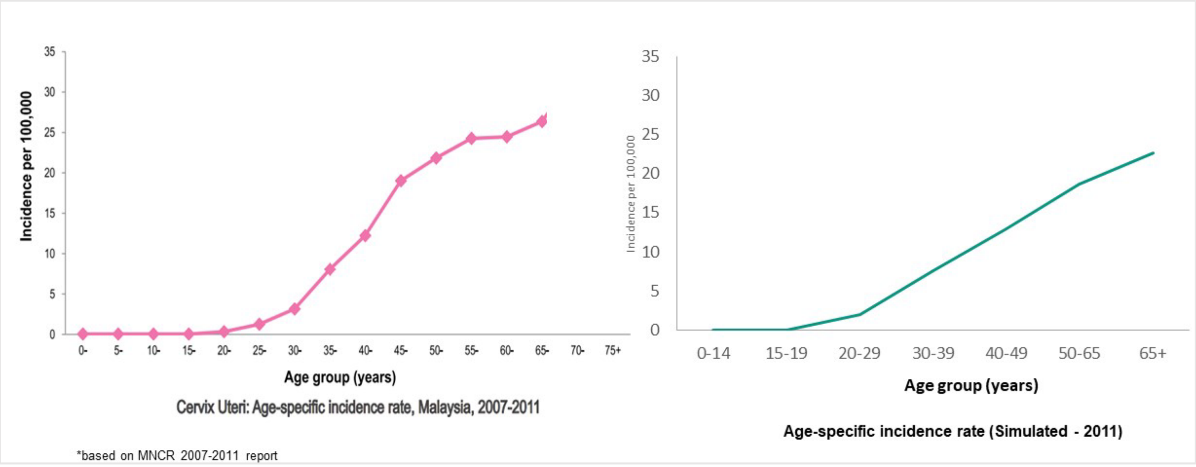

Note: MNCR=Malaysian National Cancer Registry

b) Validation: Population

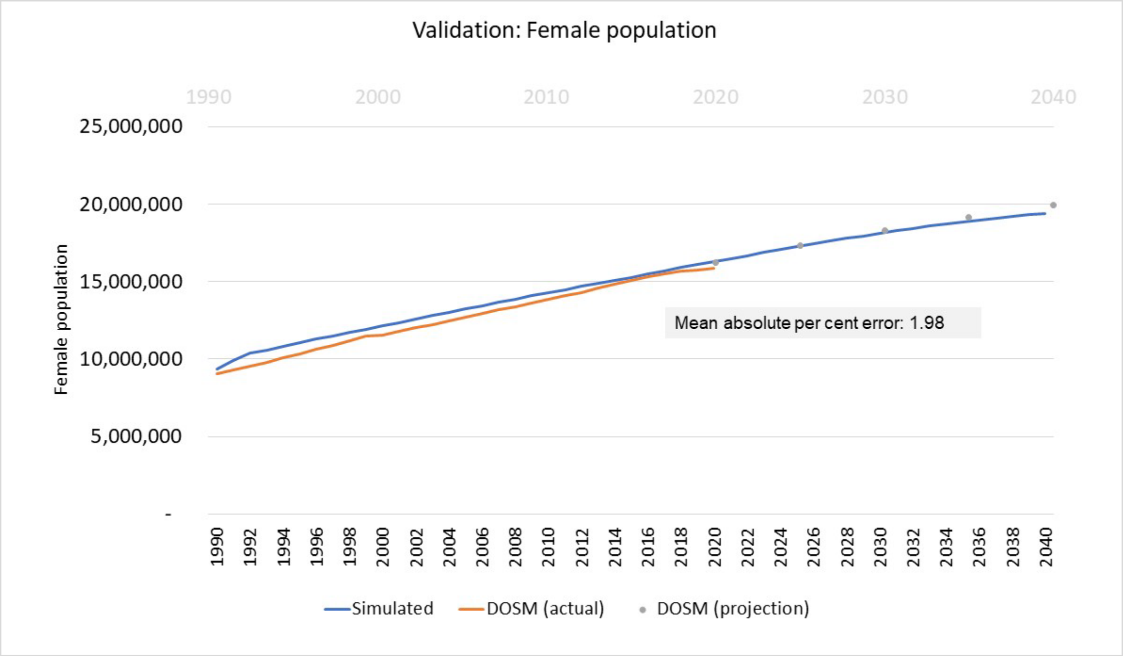

Note: DOSM=Department of Statistics Malaysia

c) Validation: Prevalence of HPV Infection

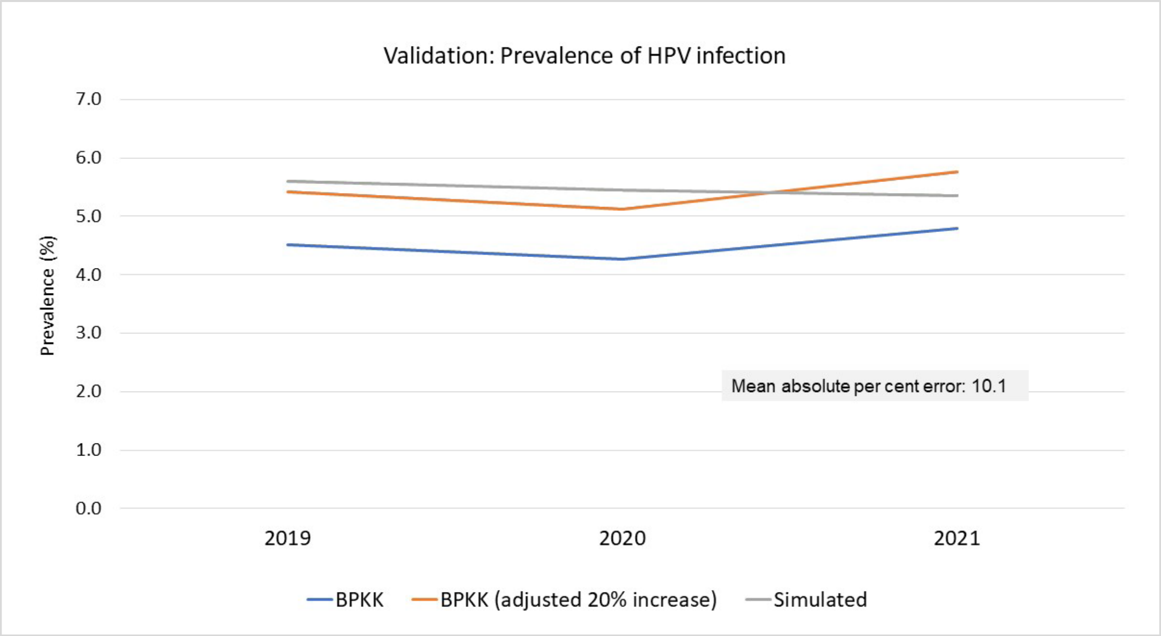

Note: BPKK=Family Health Development Division

d) Validation: Number of screenings

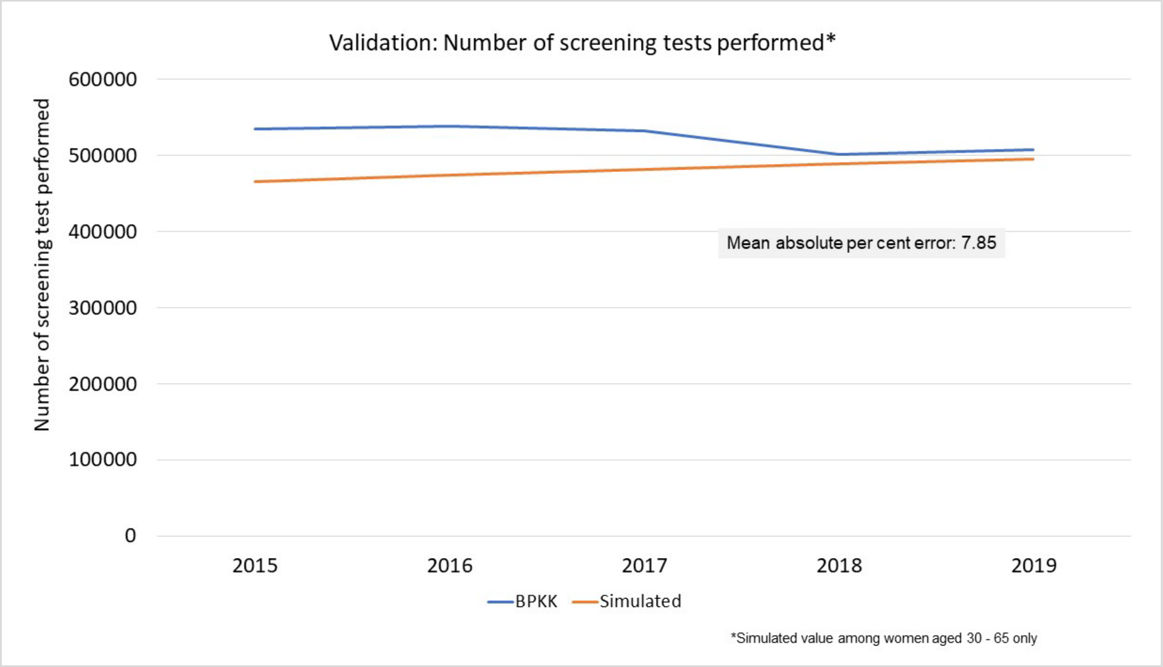

Note: BPKK=Family Health Development Division
