# Supplementary material for: Achieving cervical cancer elimination: The simulated impacts of HPV vaccination and transitioning from liquid-based cytology to HPV-based screening test
Source: PLoS One. 2024 Jul 25;19(7):e0307880. doi: 10.1371/journal.pone.0307880 (PMC11271949; doi:10.1371/journal.pone.0307880)
Supplement: S4 File — (PDF) [file pone.0307880.s004.pdf]

Appendix D: Alternative Scenarios Analysis

To address several uncertainties, Scenario A was simulated by incorporating the following alternative scenarios:

1) A lower adherence to follow-up upon a positive HPV screening test at 50 – 60% as opposed to 80 – 90%. The approach whereby 70% of women screened with HPV five-yearly was used for comparison. Figure A1 shows that at a lower follow-up rate, a four-year delay in achieving elimination was projected (The year 2056 instead of 2052).

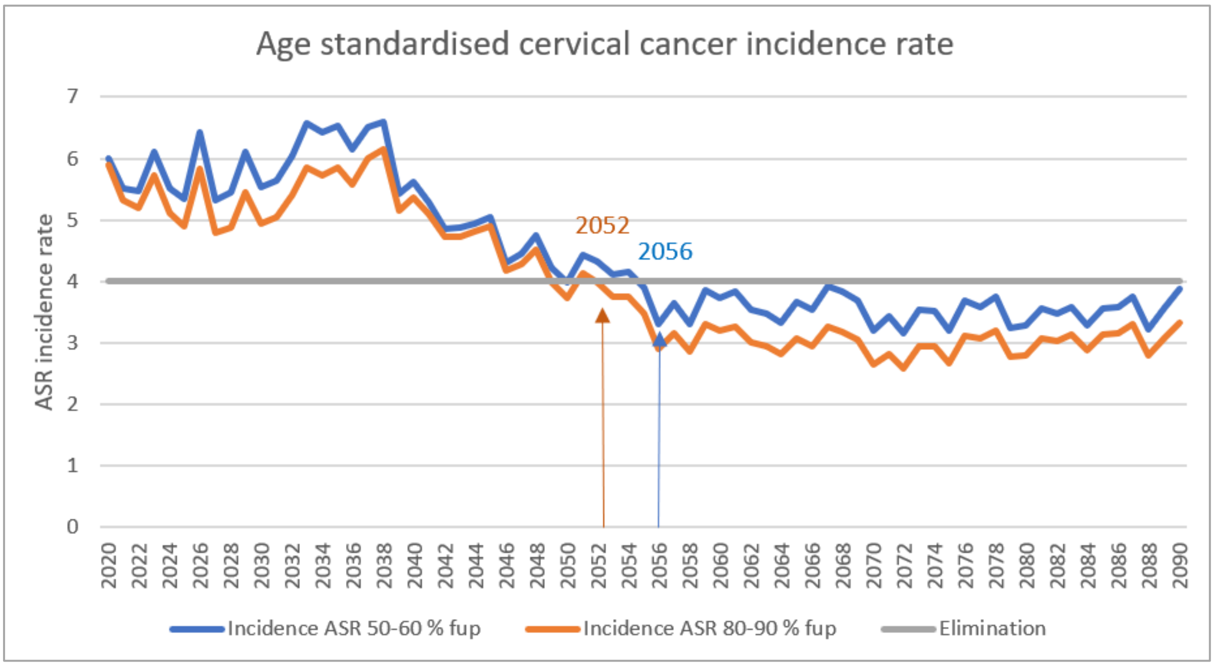

Figure A1: Age-standardised incidence rate according to different population-based HPV vaccination coverage
