# Supplementary material for: Achieving cervical cancer elimination: The simulated impacts of HPV vaccination and transitioning from liquid-based cytology to HPV-based screening test
Source: PLoS One. 2024 Jul 25;19(7):e0307880. doi: 10.1371/journal.pone.0307880 (PMC11271949; doi:10.1371/journal.pone.0307880)
Supplement: S5 File — (PDF) [file pone.0307880.s005.pdf]

Appendix E: Sensitivity Analysis

The sensitivity analyses were performed based on the baseline model in which liquid-based cytology (LBC) was used as the primary screening method and sustained at the current screening coverage of 25% of at-risk women. All other assumptions and parameters are maintained. The parameters below were tested for sensitivity analysis:

Parameters	Baseline rate	Range of rates for sensitivity analysis
Non-adherence rate (% missing follow-up upon positive HPV test)	20%	0 - 50%
Percentage infected by HPV for age group 20 - 29	0.060	0.040 - 0.0747
Percentage infected by HPV for age group 30 - 39	0.056	0.040 - 0.060
Percentage infected by HPV for age group 50 - 65	0.058	0.030 - 0.060
Unvaccinated rate (annual % girls not vaccinated)	10%	0 - 40%

Figure 1 shows the baseline scenario's tornado diagram for varying parameter rates. The projected year for cervical cancer elimination in the calibrated baseline model was 2070. The sensitivity analysis shows that the largest impact towards accelerating or bringing the elimination earlier would come from low non-adherence rates to follow-up upon positive HPV tests. At a high vaccination coverage (90%) but low screening coverage (25%) via LBC, if all women (100%) attended follow-up, the elimination is projected to be brought earlier by 17 years. In contrast, a higher unvaccinated rate of 40% would extend the elimination beyond 2090. It could also be seen that the impact of varying infection rates among the lowest age groups (20 – 29 years old) would impact the elimination more than the other age groups.

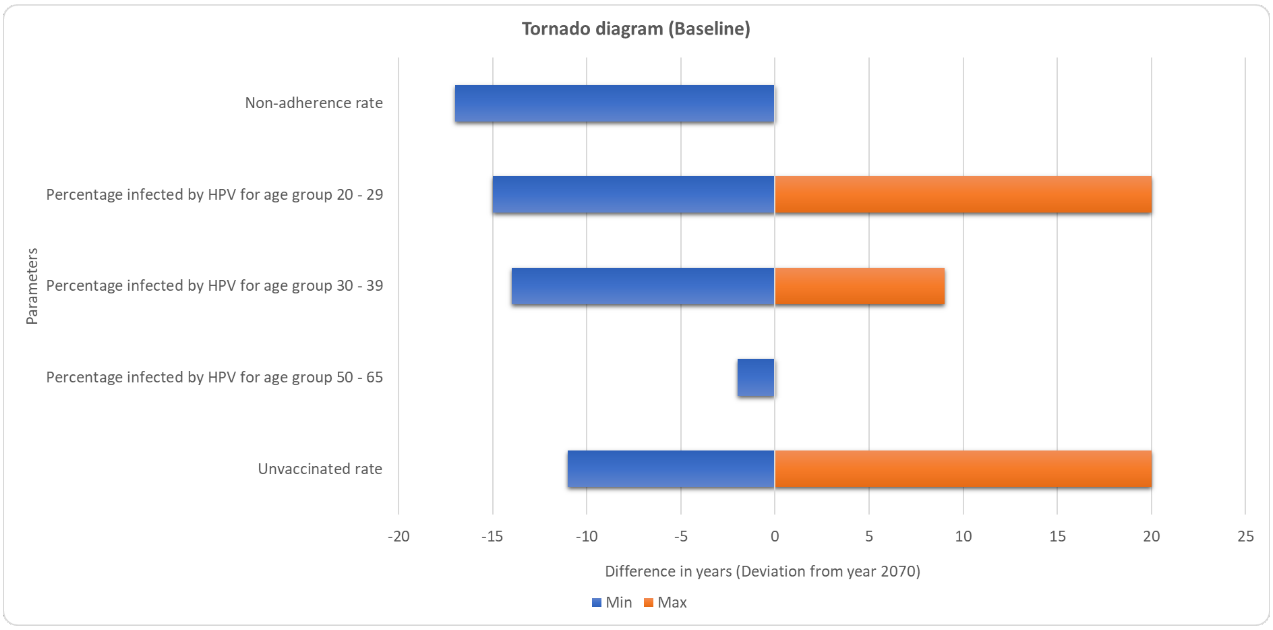

Figure 1: Tornado diagram of sensitivity analysis based on the baseline scenario
