# Supplementary material for: Achieving cervical cancer elimination: The simulated impacts of HPV vaccination and transitioning from liquid-based cytology to HPV-based screening test
Source: PLoS One. 2024 Jul 25;19(7):e0307880. doi: 10.1371/journal.pone.0307880 (PMC11271949; doi:10.1371/journal.pone.0307880)
Supplement: Attachment — Submitted filename: Response to editor and reviewers_100724.docx [file pone.0307880.s007.docx]

**Response to editor and reviewers**

**Journal Requirements:**

**Please review your reference list to ensure that it is complete and correct. If you have cited papers that have been retracted, please include the rationale for doing so in the manuscript text, or remove these references and replace them with relevant current references. Any changes to the reference list should be mentioned in the rebuttal letter that accompanies your revised manuscript. If you need to cite a retracted article, indicate the article’s retracted status in the References list and also include a citation and full reference for the retraction notice.**

Response: Thank you for your constructive feedback. We have reviewed and updated the reference list. We would like to confirm there is no citation of retracted or unpublished materials in the reference list.

**Reviewer #3:**

**With what strategy The Ministry of Health Malaysia aims to fully replace liquid-based cytology (LBC) with Human, Papillomavirus (HPV) tests and increase the screening coverage from a baseline of 25% to 40% by 2023, followed by a 10% yearly increment until 70% coverage?**

Response: Thank you for your comment. The strategy being proposed by the Ministry of Health Malaysia to replace LBC with HPV tests and increase screening coverage has been added to the introduction session, along with the relevant sources.

Change: Page 4, Line 95-98, clean version

To achieve this, the MOH plans to increase the yearly procurement of HPV test kits to replace LBC and expand screening through collaborative platforms such as private facilities, workplace screening programs, and nonprofit organizational activities [6, 13].
